# Supplementary material for: Effect of Dietary Chlorella vulgaris or Tetradesmus obliquus on Laying Performance and Intestinal Immune Cell Parameters
Source: Animals (Basel). 2023 May 9;13(10):1589. doi: 10.3390/ani13101589 (PMC10215890; doi:10.3390/ani13101589)
Supplement: Supplementary file 1 [file animals-13-01589-s001.zip › animals-2273698-supplementary.pdf]

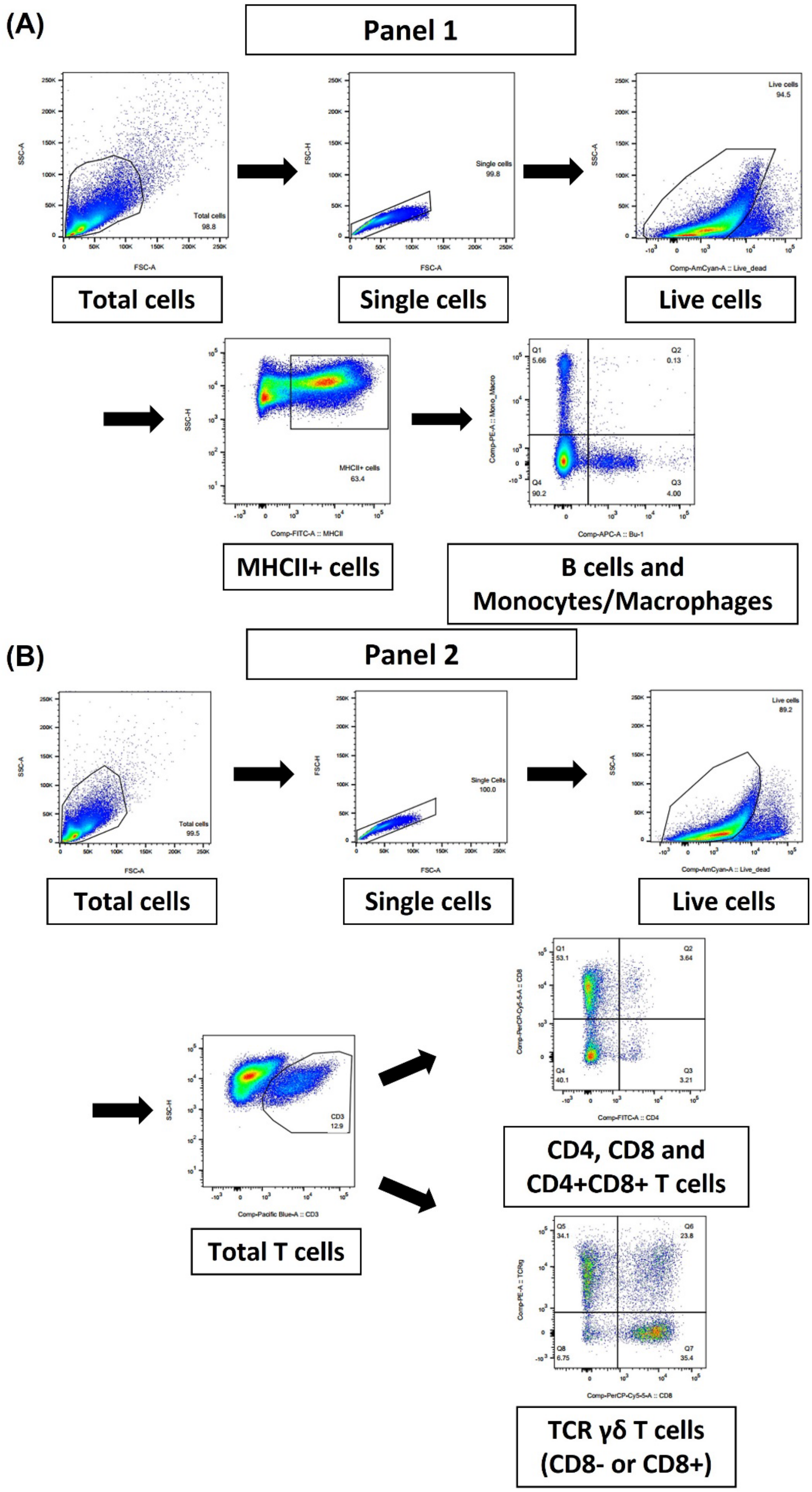

Supplementary Figure 1. Flow cytometry gating strategy in this study. All samples were analyzed single cells and live cells before main analysis. B cells and Monocytes/Macrophage were gated on panel 1 (A) and T cells were gated on panel 2 (B).
